# Editorial: Advanced nanomaterials and stem cells-based biomaterials for bone tissue engineering

**DOI:** 10.3389/fchem.2022.1065756

**Published:** 2022-11-04

**Authors:** Xibo Pei, Xue Yuan, Junyu Chen

**Affiliations:** ^1^ Department of Prosthodontics, State Key Laboratory of Oral Diseases, National Clinical Research Center for Oral Diseases, West China Hospital of Stomatology, Sichuan University, Chengdu, China; ^2^ Department of Otolaryngology-Head & Neck Surgery, School of Medicine, Indiana University, Indianapolis, IN, United States; ^3^ Kennedy Institute of Rheumatology, University of Oxford, Oxford, United Kingdom

**Keywords:** nanomaterials, bone tissue engineering, stem cells, drug delivery, implant

## Nanomaterials

Metal nanoparticles (MNPs) have piqued the interest of researchers because they offer significant advancements, particularly in the effective delivery of therapeutic drugs and the prevention of multidrug resistance. In the hard tissue engineering field, MNPs exhibit powerful bone regeneration potential, pro-angiogenic activity, antibacterial properties, and unique drug delivery capacity. Liu et al. reviewed MNPs for treating bone disease and regeneration. The authors began by introducing MNP classifications and three commonly used methods for drug loading. Applications of MNPs in bone regeneration, osteoarthritis, osteomyelitis, and bone tumors were summarized in detail. Finally, the authors pointed out some of the biggest problems that need to be solved before MNPs can be used in clinical settings.

The rapid growth of osteoporotic patients with dentition defects poses significant challenges for implant material technology. Vascularization is vital in the early stage of bone regeneration, hence implants with designs aimed at rescuing the compromised osteoporotic angiogenic microenvironment are optimal. Yan et al. found that 0.2–1 mM strontium ion could promote vascular endothelial growth factor A (VEGFA) and angiopoietin-1 (Ang-1) expression in a co-culture system with human umbilical vein endothelial cells (HUVECs) and bone marrow mesenchymal stem cells (MSCs). Then, a titanium surface with zinc and strontium was manufactured through plasma electrolytic oxidation, which was consequently demonstrated to exhibit increased osteogenic capacity *in vitro* and stronger osseointegration when subjected to comprehensive assessment *in vivo*. These results provide solid evidence that doping titanium implants with a combination of zinc and strontium is an ideal modification method to optimize the titanium implant’s properties. With further research into the function and mechanism of bioactive components at implanting sites, it is promising to construct bioactive multifunctional implants for clinical applications.

Excellent biocompatibility and biodegradability are important prerequisites for biomaterials. Xu et al. evaluated the biodegradability and biocompatibility of a hot-extruded zinc-copper-iron (Zn-Cu-Fe) alloy. Zn-Cu-Fe showed a faster degradation rate than that of Zn. The cytotoxicity was demonstrated to be acceptable by live and dead cell staining, metabolic activity determination, and lactate dehydrogenase release detection with cells. In terms of hemocompatibility, Zn-Cu-Fe activated coagulation and complement systems but had no significant effect on erythrocyte, platelet, or leukocyte numbers. Their results highlighted Zn-Cu-Fe alloy as a potential biodegradable material for craniomaxillofacial implants. Further research will be needed to test it directly on the craniomaxillofacial injury sites.

Hydrogels provide a scaffold for the safe delivery of therapeutic cells to the wound site while shielding the delivered cells from immune attack. Nowadays, increasing hydrogel scaffolds that mimic the structure and composition of bone tissue have emerged. Sun et al. fabricated a novel gelatin/carboxymethyl chitosan (CMC)/nano-HA/β-TCP osteogenic scaffold with highly connected macropores. The authors provided a comprehensive assessment regarding the biocompatibility and osteoinductivity of this scaffold. The scaffold showed no cytotoxicity and enhanced osteoinduction and osteoconduction. This is an exciting discovery because, although being a four-component scaffold, the manufacturing method is straightforward, and the combination has a greater effect on bone formation than any individual component.

MicroRNAs (miRNAs) play a critical role in osteogenic differentiation. But their instability in serum and poor transfection efficiency limited their use in tissue engineering. Nano-delivery systems have been employed to overcome these drawbacks of naked miRNAs. Qin et al. conjugated cationic polymer polyethylenimine (PEI) to polyethyleneglycol (PEG)-modified graphene oxide (GO) for miR-29b loading and delivery. The miR-29b/GO-PEG-PEI complex, with good biocompatibility, prominent miRNA loading ability, and significant cell transfection efficiency, was then encapsulated in a chitosan (CS) hydrogel. In a skull defect model, the composite hydrogel demonstrated remarkable osteogenic activity. These results demonstrate that GO-based nanomaterials are promising for gene delivery and could be employed to promote bone formation.

## Stem cells-based biomaterials

MSCs have long been used for bone tissue engineering. Multiple mesenchymal stem cell sources have been reported. Common ones include bone marrow and fat. Harvesting bone marrow MSCs has limitations, including the limited number of MSCs, pain, and the risk of morbidity associated with the bone marrow aspiration procedure. It has been shown that a large number of adipose-derived MSCs (ADSCs) can be easily isolated from adipose tissue using a minimally invasive procedure. Zhao et al. explored the effect of adipose-derived mesenchymal stem cells (ADSCs) on tendon injury and the underlying mechanism. Injecting ADSCs into damaged tendon tissues accelerated tissue regeneration *in vivo*. ADSCs promote tenocyte proliferation by stimulating lncRNA Morf4l1 expression. Tendons are the special connective tissue that connects bones to muscles, and the healing of tendon injuries is still a challenge. The findings reported by Zhao et al. provide support for stem cell engineering therapy of tendon injury.

Periodontitis is characterized by bacterial infection, inflammatory reactions, and periodontal degeneration. In severe cases, periodontal disease causes extensive bone loss surrounding teeth, increasing the chances of tooth loss. Therefore, there is a great need for the development of a tissue engineering approach for the regeneration of alveolar bone. Periodontal ligament stem cells (PDLSCs) can regenerate alveolar bone, but their function is compromised in the inflammatory environment. According to Sun et al., exosomes derived from human gingival mesenchymal stem cells (GMSC-Exo) could attenuate the inflammatory response of PDLSCs. GMSC-Exo was extracted from the culture supernatant of GMSCs by ultracentrifugation. GMSC-Exo suppressed NF-κB signaling and Wnt5a expression in PDLSCs, hence creating a regenerative microenvironment for periodontal tissues. Exosomes, derived from mesenchymal stem cells, have been widely studied in the treatment of many diseases and have emerged as innovative tools for the development of nanomedicine. Exosome therapy, unlike stem cell therapy, does not utilize living cells, generating fewer immediate safety concerns. Exosomes are also simpler and less expensive to produce and manipulate than stem cells, making them an appealing alternative for stem cell therapy.

